# Real-world efficacy and safety of mobocertinib in *EGFR* exon 20 insertion-mutated lung cancer

**DOI:** 10.3389/fonc.2022.1010311

**Published:** 2022-09-20

**Authors:** Waleed Kian, Petros Christopoulos, Areen A. Remilah, Esther Levison, Elizabeth Dudnik, Walid Shalata, Bilal Krayim, Ranin Marei, Alexander Yakobson, Martin Faehling, Dolev Kahala, Inbal Sara Granot, Dina Levitas, Nir Peled, Laila C. Roisman

**Affiliations:** ^1^ The Institute of Oncology, Shaare Zedek Medical Center, Jerusalem, Israel; ^2^ Department of Thoracic Oncology, Thoraxklinik at Heidelberg University Hospital, Member of the German Center for Lung Research (DZL), and Translational Lung Research Heidelberg, Heidelberg, Germany; ^3^ Medical School for International Health, Ben-Gurion University of the Negev, Beer-Sheva, Israel; ^4^ Thoracic Cancer Service, Assuta Medical Centers, Ben-Gurion University, Tel-Aviv, Israel; ^5^ The Legacy Heritage Oncology Center and Dr. Larry Norton Institute, Soroka Medical Center and Ben-Gurion University, Beer-Sheva, Israel; ^6^ Department of Pneumology, Esslingen Hospital, Esslingen, Germany

**Keywords:** mobocertinib, nsclc, lung cancer, EGFR exon 20 insertion mutation, Real World Data

## Abstract

**Background:**

Non-small cell lung cancer (NSCLC) harboring *EGFR* exon 20 insertions (*EGFR*ex20ins) is relatively resistant to the existing *EGFR* tyrosine kinase inhibitors (TKIs). Mobocertinib is a novel TKI that selectively targets *EGFR*ex20ins and has demonstrated therapeutic efficacy in pretreated patients with tumors harboring these mutations.

**Methods:**

This is a retrospective, non-interventional, multicenter real-world study aimed at assessing the efficacy and safety of mobocertinib in patients with EGFRexon20ins who received 160 mg QD monotherapy as part of expanded access. Data collection was based on patients’ records. PET-CT or CT scans were used to measure systemic response, while brain MRIs were used to examine intracranial response as part of the follow-up.

**Results:**

16 patients were included in this report. Mobocertinib was administered to 31.3% (5) of patients as first-line, 50% (8) as second-line, and 18.7% (3) as a later-line therapy. The median age was 65 years (range, 38-83), 75% (12/16) were female, and 50% (8/16) had brain metastases at baseline before mobocertinib treatment. The objective response rate (ORR) to mobocertinib was 25% (4/16) (1/5 for first line and 3/11 for other lines), disease control rate (DCR) was 75% (12/16) with a follow-up period of 11 months. The median duration of treatment (mDoT) was 5.6 months across all patients, and 8.6 months in responders. Based on the presence or absence of brain metastasis, the mDoT was 14.8 and 5.4 months (p=0.01), respectively. Mobocertinib Grade ≥3 treatment-related adverse events (TRAEs) included diarrhea (19%), nausea (6%) and renal failure (6%). Dose reduction was reported in 25% of cases to 80 mg.

**Conclusion:**

Mobocertinib in compassionate use exhibited an ORR of 25%, which is very similar to that of the phase 2 EXCLAIM study and clearly better than historical data of monochemotherapy or conventional EGFR inhibitors. The greatest benefit was noted in patients without brain metastases, who showed durable effects with mDoT 14.8 months, while intracranial activity was limited. These findings may assist therapeutic considerations, inasmuch as results from the EXCLAIM cohort-3 dedicated to brain lesions are not available yet.

## Highlights

Real-world data for 16 patients with *EGFR*ex20ins-positive NSCLC treated with compassionate mobocertinib, 31.3% of patients first-line.
Mobocertinib showed systemic efficacy with an ORR of 25%.
Brain activity was limited
Grade ≥3 TRAEs included diarrhea (19%), nausea (6%) and renal failure (6%).
Dose reduction rate was 25%.

## Introduction

Epidermal growth factor receptor (*EGFR*) is an oncogenic transmembrane protein that upon activation by specific ligands stimulates intrinsic intracellular tyrosine kinase activity and elicits downstream signaling (1, 2). These downstream signaling processes enable DNA synthesis, cell proliferation, angiogenesis, inhibition of apoptosis and modulate cell migration, adhesion and invasion (2, 3). Mutations that cause *EGFR* upregulation are associated with cancer, with *EGFR* mutations present in 12-38% of lung adenocarcinomas, the main form of non-small cell lung cancer (NSCLC) (4). *EGFR* exon 20 insertion (*EGFR*ex20ins) mutations occur in 4-12% of *EGFR*-mutated NSCLCs and approximately 2% of all NSCLCs (5, 6).

*EGFR* exon 19 deletions and *EGFR* exon 21 p.L858R, together representing up to 80-85% of *EGFR*-activating mutations, are highly sensitive to standard *EGFR* tyrosine kinase inhibitors (TKIs) such as osimertinib (7–9). The less common *EGFR* exon 20 insertion (*EGFR*ex20ins) mutated NSCLCs are challenging to treat due to their relative insensitivity to the standard *EGFR*-TKIs, with a response rates of 0-10% and median progression-free survival (PFS) of 1-3 months in several retrospective analyses. *EGFR* exon 20 insertions are driver mutations comprised of a variety of in-frame insertions that constitutively upregulate *EGFR* kinase activity (6). Their *de-novo* resistance to first and second-generation tyrosine kinase inhibitors arises from the conformational changes to the receptor and modified structure of the kinase domains, preventing the binding of TKIs to the active site (4, 6). Comprehensive genomic sequencing and algorithmic screening reveal a broad variety of *EGFR* exon 20 insertions: insertions and/or duplications of 3-21 base pairs, confined to the proximal 5’ end of the exon between codons 763 and 775 and demonstrating wide heterogeneity at the amino acid level (6, 10, 11).

The clinical and pathological aspects of *EGFR*ex20ins-positive NSCLC obtained from a small number of reports appear to be similar to those of common *EGFR* mutations. Patients with *EGFR*ex20ins-positive NSCLC were more prevalent among nonsmokers (p<0.0001), with no significant differences in gender, age, ethnic origin or stage at diagnosis (10).

Platinum-based chemotherapy is the first-line treatment for *EGFR*ex20ins-positive patients, however progressive disease (PD) often develops within 6 months (5, 12). Upon disease progression, the FDA recently approved second-line therapy with the bispecific *EGFR*-*MET* monoclonal antibody amivantamab and the novel *EGFR* exon20-directed TKI mobocertinib (5, 13–15). First and second generation TKIs have limited efficacy in treating *EGFR*ex20ins-positive NSCLC, however several next generation *EGFR* TKIs have shown pre-clinical activity against this unique subset of mutation and are currently in development, for example DZD9008 and CLN-081 (16, 17).

Mobocertinib is an oral, irreversible, novel TKI that selectively targets in-frame *EGFR*ex20ins mutations in NSCLC (5). A 2021 phase 1/2 open-label, non-randomized clinical trial has demonstrated clinically benefit, with an overall response rate (ORR) of 28% per independent review committee (IRC), median duration of response (DoR) of 17.5 months, median PFS of 7.3 months and median overall survival (OS) of 24 months. The safety profile is manageable, and the most common treatment-related adverse events were diarrhea and rash. A multicenter, open-label clinical trial that led to FDA accelerated approval demonstrated mobocertinib to have a weighted response rate nearly 3 times higher than standard of care, with the aforementioned PFS and OS for mobocertinib comparing to 3.3 months and 12.4 months respectively in a real-world group (14). Mobocertinib has demonstrated activity against common and uncommon activating mutations, with limited off-target activity, and 2-133 times greater potency against *EGFR*ex20ins than erlotinib, gefitinib, afatinib or osimertinib (5, 18).

This multicenter study is based on international data for patients with *EGFR*ex20ins-positive NSCLC treated with mobocertinib.

## Methods

### Study design

This is a retrospective, non-interventional, multicenter real-world study that aims to assess the efficacy and safety of the treatment in patients with *EGFR*exon20ins with an emphasis on the treatment with mobocertinib in *EGFR*ex20ins-positive patients.

The primary endpoint of this retrospective data analysis was the objective response rate (ORR) - the proportion of patients who had a complete response (CR) or a partial response (PR) as assessed by investigator RECIST v1.1 criteria. The secondary endpoints were: i) assessment of treatment-related adverse events (TRAEs) determined by the treating physician; ii) disease control rate (DCR) defined as the proportion of patients with a CR, a PR and stable disease (SD); iii) median duration of treatment (mDoT) defined as the time between the first dose and the last dose or death, regardless of cause of death); and iv) median overall survival (mOS) defined as the time between the date of diagnosis of metastatic or advanced stage lung cancer and death.

### Study population and treatment

This information was obtained by oncological centers that specialize in the treatment of lung cancer patients. Histologically confirmed NSCLC at a locally advanced or metastatic stage, an age of ≥18 years, a confirmed *EGFR*ex20ins, and availability for at least one follow-up assessment of response by computed tomography (CT) scan and/or magnetic resonance imaging (MRI) were all required for inclusion.

Next generation sequencing (NGS)-based genomic profiling, polymerase chain reaction (PCR) or Sanger sequencing from tissue and/or liquid biopsy were all acceptable testing techniques for detecting *EGFR*exon20ins mutations.

Most *EGFR*ex20ins-positive patients were treated with mobocertinib, which was given orally once a day at a dose of 160 mg. Reduced initial doses, dosage reductions and re-escalations were chosen at the discretion of treating physicians. Mobocertinib treatment was continued until disease progression, a lack of therapeutic benefit, intolerable toxicity, the patient’s withdrawal of consent or a decision by the treating physician.

### Data collection

Clinical features and treatment data were obtained from the medical records, anonymized and transmitted for statistical analysis by the treating physicians. Patients’ demographics and clinical characteristics (gender, date of birth, ethnicity, smoking and ECOG performance status, disease stage, previous treatments, histology, *EGFR*ex20ins mutation status, testing method and co-mutations), treatment choice (duration and dose, best response, as well as date, type and location of progression) and safety data were included in the data.

### Ethics statement

The study protocol was approved by the ethics committee of the Shaare Zedek Medical Center (approval no. 0432-20-SZMC) and other participating institutions. Informed consent for each patient was not necessary for this retrospective analysis.

### Statistical analysis

The Kaplan-Meier technique and a confidence interval (CI) of 95% were used to evaluate DoT and OS. For PFS, patients who are alive at the time of data cut-off and have no documented progression have been censored at the time of data cut-off or last contact, while patients who started additional anticancer therapy in the absence of proven PD (e.g., because of an adverse event) were censored at the moment of treatment termination. For OS, patients who were lost to follow-up or still alive at the time of last contact were censored at the date of last contact. A log-rank test with a level of significance of 5% (chi square p=0.05) was performed to compare subgroups.

## Results

### Patients

Data from 16 patients with locally advanced or metastatic NSCLC *EGFR*ex20ins-positive by tissue NGS, treated between May 2017 and June 2022, were retrospectively analyzed. Their demographics, clinical and pathological characteristics, and mutations subtype with their co-mutations are presented in **Tables 1** and **2**. The median age was 65 years (range, 38.3-83.6). More patients were female (75%). Whereas 56% of patients never smoked, the proportion of former smokers was 37% and 7% were currently smoking. Overall, 81% of patients presented with a good (0 to 1) ECOG performance status. Prior to the use of mobocertinib, 50% of patients had brain metastasis. First line treatment with mobocertinib was received by 31.3% of patients, second line treatment by 50% of patients and third line treatment by 18.7% of patients.

**Table 1:** Demographics and characteristics of patients prior to mobocertinib administration.

Characteristics	N = 16
**Age**	
Median (range), years	65 (38-83)
**Gender, n (%)**	
Male	4 (25)
Female	12 (75)
**Smoking history, n (%)**	
Former	6 (37.5)
Never	9 (56.3)
Current	1 (6.2)
**Histology, n (%)**	
Adenocarcinoma	16 (100)
**Stage of disease at diagnosis, n (%)**	
IV	16 (100)
**PD-L1 status, n (%)**	
>50%	1 (6.3)
1-49%	5 (31.3)
<1%	8 (50)
N/A	2 (12.4)
**ECOG, n (%)**	
0	8 (50)
1	5 (31.3)
2	2 (12.4)
N/A	1 (6.3)
**Brain metastasis before Mobocertinib, n (%)**	
No	8 (50)
Yes	8 (50)
**Mobocertinib line of treatment, n (%)**	
1^st^	5 (31.3)
2^nd^	8 (50)
3^rd^	3 (18.7)
**EGFRex20ins mutation, n (%)**	
A763_Y764insFQEA	1 (6.3)
A767_V769dup	1 (6.3)
V769_D770insASV	2 (12.5)
D770_N771insG	1 (6.3)
D770_N771insSVD	1 (6.3)
D770_N771insGL	1 (6.3)
D770_P772dup	1 (6.3)
D770delinsDGP	1 (6.3)
D770delinsDNPH	1 (6.3)
N771_H773dup	2 (12.5)
N771>GY	1 (6.3)
H773_V774insAH	1 (6.3)
H773_V774delinsLM	1 (6.3)
N/A	1 (6.3)

ECOG, Eastern Cooperative Oncology Group; N/A, not available/not applicable; PD-L1, Programmed death-ligand 1; EGFRex20ins, EGFR exon 20 insertion; RT, radiotherapy.

**Table 2:** Other co-mutation found with EGFRex20ins.

Other co-mutations with allele frequency	EGFRex20ins mutation
TP53 V173M 90.2%, EGFR amp 7.66	A763_Y764insFQEA
TP53 H214R 0.5%, TP53 G334fs 0.9%	A767_V769dup
MYC rearrangement intron 1	V769_D770insASV
TP53 A288fs*57 37.5%	V769_D770insASV
None	D770_N771insSVD
TP53 T163A 36.6%	D770_N771insG
None	D770_P772dup
None	D770_N771insGL
TP53 H115fs*34 20.4%	D770delinsDGP
None	D770delinsDNPH
EGFR T638M 0.1%	N771_H773dup
BRCA2 S1982Rfs*22 48.54%, TP53 A159V 62.06%, SETD2 Q1324* 66.59%	N771_H773dup
None	N771>GY
None	H773_V774insAH
EGFR amplification 9.07	H773_V774delinsLM

### Efficacy

#### Response

**Figure 1:**
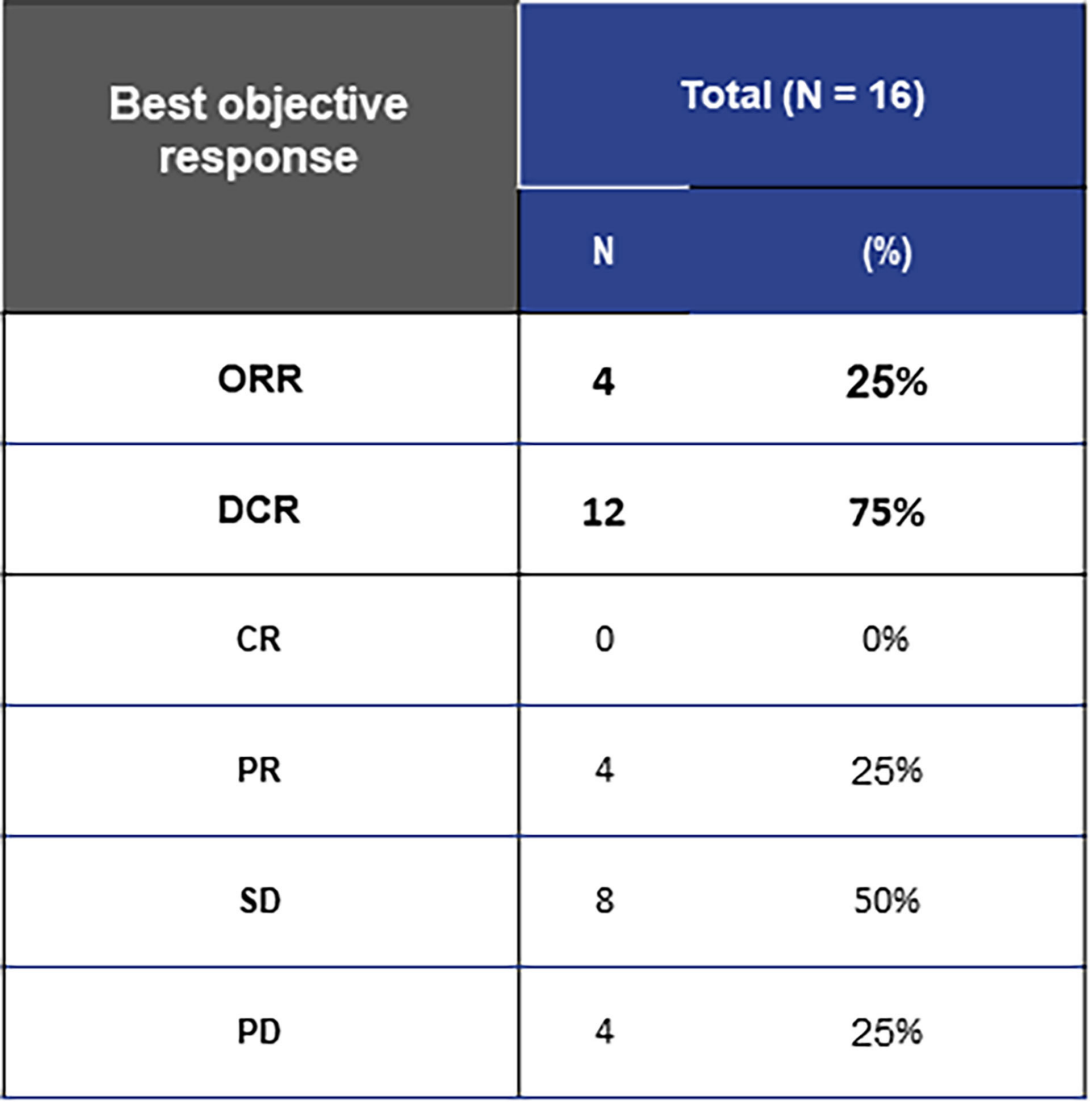
Efficacy of mobocertinib in *EGFR*ex20ins-positive patients overall.

**Figure 2:**
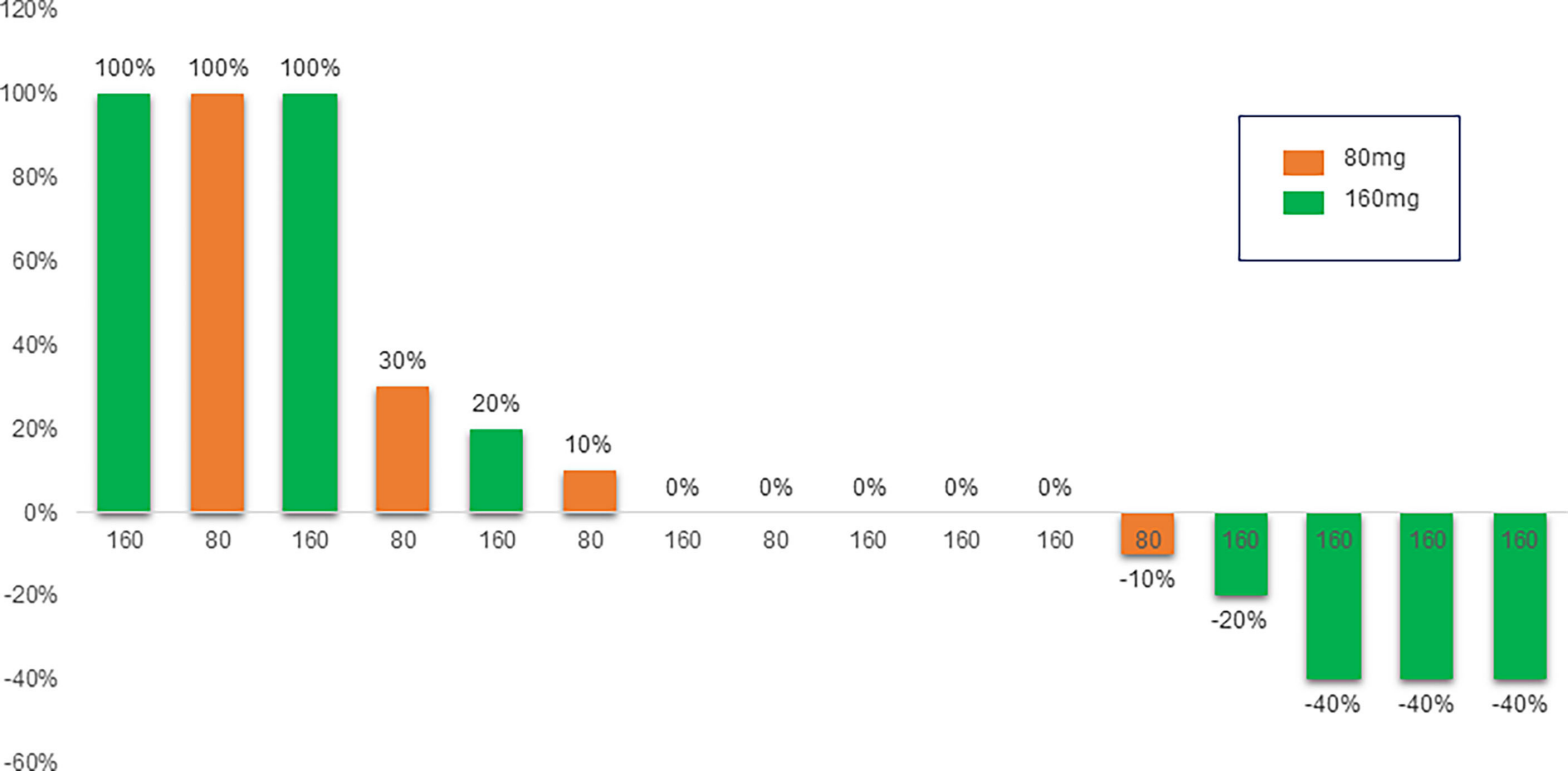
The best response to mobocertinib from baseline in target lesions.

The efficacy results are presented in **Figure 1**. The overall ORR was 25%, with 4 patients (25%) having a PR and no documented CR. Non-responders included 4 patients (25%) showing PD and 8 (50%) showing SD. The overall DCR was 75% with a median follow-up of 11 months. The best response to mobocertinib from baseline in target lesions is shown in **Figure 2**; five patients (31.3%) had no measurable target lesion.

### Duration of treatment

**Figure 3:**
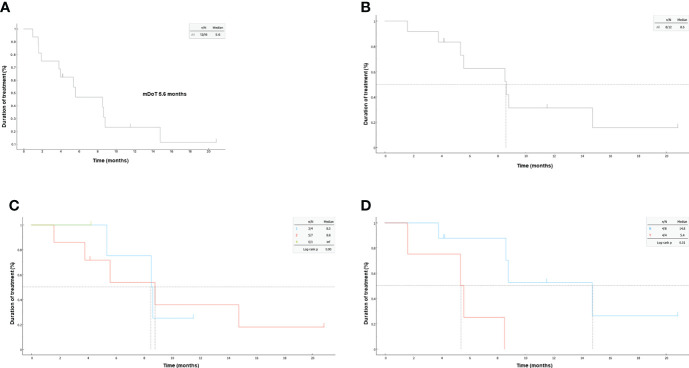
Kaplan-Meier plots of duration of treatment (DoT); **(A)** in the overall population and **(B)** according to brain involvement. **(C)** for responders according to line of treatment, **(D)** for responders according to brain metastasis.

The mDoT across all patients was 5.6 months, and 8.6 months in responders (**Figures 3A, B**). Among responders, there was no difference in mDoT between the first or second line of treatment with 8.5 and 8.8 months, respectively (p=0.9, **Figure 3C**). Notably, the mDoT was 14.8 and 5.4 months (p=0.01), based on the presence or absence of brain metastasis, as illustrated in **Figure 3D**.

### Safety

The TRAEs that occurred at any grade are presented in **Table 3**. Most TRAEs were of low severity (grade ≤2). Diarrhea was the most common TRAE (n=9, 52%), 6 of which were grade ≤2, followed by nausea (n=4, 25%), rash (n=2, 12.5%) and renal failure (n=2, 12.5%). Grade ≥3 TRAEs included diarrhea (n=3, 19%), nausea (n=1, 6.3%) and renal failure (n=1, 6.3%). Dose reduction occur in 25% mainly due to diarrhea.

**Table 3:** Treatment-related adverse events (TRAEs) that occurred at any grade in patients treated with mobocertinib (N=16).

Adverse Events	Grade 1-2	Grade 3	Grade 4	Grade 5
Fatigue	1 (6%)	0	0	0
Nail toxicity	0	0	0	0
Rash	2 (12%)	0	0	0
Dry skin	1 (6%)	0	0	0
Decrease appetite	1 (6%)	0	0	0
Diarrhea	**6 (38%)**	**3 (19%)**	0	**0**
Headache	0	0	0	0
Pruritis	1 (6%)	0	0	0
Nausea	3 (19%)	1 (6%)	0	**0**
Thrombocytopenia	0	0	0	0
Leukopenia	0	0	0	0
Cough	0	0	0	0
Constipation	0	**0**	0	**0**
Acne	1 (6%)	0	0	0
Anemia	1 (6%)	0	0	0
Stomatitis	0	0	0	0
Renal Failure	1 (6%)	0	1 (6%)	0

## Discussion

In April 2020, mobocertinib (Exkivity, TAK-788, Takeda Pharmaceuticals, Inc.) was granted Breakthrough Therapy Designation from the FDA based on preliminary results of the phase 1/2 EXCLAIM study demonstrating an ORR of 43% assessed by investigator and long-term benefit (19). On September 15th 2021, mobocertinib received accelerated FDA approval for the second-line treatment of locally advanced or metastatic NSCLC in *EGFR*ex20ins-positive adult patients with disease progression after prior platinum-based chemotherapy (20). However, a later update reported the confirmed ORR as 28% per assessment of the independent review committee (IRC), and 35% per assessment by the investigators. Our data show very similar efficacy of mobocertinib to that reported by the IRC, with ORR, DCR, and mDoT 25%, 75% and 5.6 months respectively, comparing to 28%, 78%, and 7.3 months respectively for the platinum-pretreated patient (PPP) subset of the EXCLAIM (5). Of note, even though our analysis included some first-line patients, these were exclusively treated within the compassionate-use program (CUP), while patients treated within trials, including the ongoing first line EXCLAIM-2 trial, were excluded. Since patients could be enrolled already from the first line in the CUP only if deemed unsuitable for chemotherapy, our patients correspond rather to the pretreated population of the EXCLAIM study. For example, no difference in mDoT between the first and second-line treatment was noted in our study (p=0.9), and overall, the efficacy and safety results were comparable to those of the EXCLAIM. Of note, the characteristics of our patients were also very similar to these of the subset of EXCLAIM, with a very similar median age (65 years vs. 60 years in PPP), as well as a predominance of women (75% vs. 66% in PPP) and never smokers (56.3% vs. 71% respectively). At the same time, our population included slightly more patients with an ECOG performance status ≥2 (6.3% vs 0% in PPP) and baseline brain metastasis (50% vs 35% in PPP).

In the EXCLAIM and PPP cohorts, all patients were required to have brain radiotherapy before starting mobocertinib. Unfortunately, the brain was the primary location of disease progression in 38% (22/58) of all PD patients and 68% (17/25) of PD patients with baseline brain metastasis (5). In our study there was no intracranial response among the 8 patients who had brain disease, and the mDoT varied widely according to whether brain metastases were present or not at baseline with 14.8 vs. 5.4 months, respectively (p=0.01). Therefore, it seems that mobocertinib has very limited brain activity. This is potentially valuable evidence that may assist therapeutic considerations in patients with EGFRex20ins tumors, inasmuch as results from the cohort-3 of EXCLAIM dedicated to measurable brain lesions are not available yet.

However, it should be noted that a worse outcome for NSCLC with EGFR exon20 insertions and brain metastases has also been described under conventional therapies, i.e. platinum-based chemotherapy and EGFR inhibitors (21), therefore brain involvement appears to be an adverse prognostic factor for this patient group under several different therapies. Thus, use of mobocertinib in patients with brain metastases will require complementary use of radiotherapy in order to extend the intracranial PFS, as is also standard for patients with other oncogene-driven NSCLC beyond the first line (22). At the same time the ORR under mobocertinib was clearly superior to that of current alternatives, such as monochemotherapy and EGFR inhibitors, which both showed ORR of 0-10% in real-world studies of pretreated patients with *EGFR*exon20ins (21).

Interestingly, patients who received mobocertinib as first or second-line therapy after EGFR TKI, chemotherapy, or immunotherapy had similar confirmed ORRs and mDoT. The observed EGFRex20ins variants reflected the expected molecular diversity of variants, with a predominance of two commonly observed variants (ASV, and SVD) (23). In our report, responses were observed across all EGFRex20ins mutation subtypes, regardless of mutation frequency or position, indicating no clear genotype-activity relationship. Vasconcelos et al. found in 16 patients that the EGFR-A763 Y764insFQEA mutation is sensitive to first-, second-, and third-generation EGFR-TKI, as well as mobocertinib and poziotinib (24). One of our patients had this subtype variant and was treated with mobocertinib as first line therapy, resulting in systemic disease response and brain stable disease.

Besides, mobocertinib showed a manageable safety profile and low discontinuation rates in our real-world study, with mainly low-grade TRAEs of diarrhea, nausea, and rash. TRAEs grade 3-4 were reported in 33% of our cohort. The most common TRAE in our cohort was diarrhea, grade 1-2 and grade 3 experienced by 37.5% and 19% of patients respectively. In the PPP cohort, 96% of patients experienced at least one gastrointestinal toxicity with diarrhea being the most common, observed in 93% of patients with grade ≥3 diarrhea occurring in 22% (5). Diarrhea led to dose reduction in 11% of patients and treatment discontinuation in 4% of patients (25).

## Conclusions

Mobocertinib is a novel drug with limited efficacy for pretreated *EGFR*ex20ins-positive NSCLC, which however is clearly better than that of alternative options, like EGFR inhibitors or docetaxel. The greatest benefit was noted in patients without brain metastases, who showed durable effects with mDoT 14.8 months, while intracranial activity was limited. This real-world evidence supports previously phase 1/2 findings and highlights the potential role of brain involvement in therapeutic considerations, while results of the “brain” EXCLAIM cohort 3 and first line EXCLAIM-2 trial, as well as the development of further exon20-directed drugs are eagerly awaited.

## Limitations and biases

This retrospective analysis is certain to have several limitations, including selection bias, reporting bias, and information bias. In particular, even though our analysis included some first-line patients, these correspond rather to the pretreated population of the EXCLAIM study because they were treated within the compassionate-use program, which could enrollpatients from the first line only if deemed unsuitable for chemotherapy. Furthermore, due to the limited sample size of some subgroups, only descriptive effectiveness results were presented. Furthermore, intrinsic limits to clinical normal practice in each participating institution should be recognized, particularly in terms of *EGFR*ex20ins testing methods, intervals of radiographic assessments, and national or hospital-based treatment standards. With these constraints in mind, the data presented here corroborate the efficacy and safety findings of EXCLAIM in the real-world setting and highlight the potential role of brain involvement in therapeutic considerations, while results from the EXCLAIM cohort-3 dedicated to measurable brain lesions are still pending.

## Data availability statement

The raw data supporting the conclusions of this article will be made available by the authors, without undue reservation.

## Ethics statement

The studies involving human participants were reviewed and approved by approval no. 0432-20-SZMC. Written informed consent for participation was not required for this study in accordance with the national legislation and the institutional requirements.

## Author contributions

WK: Writing – Original Draft, Visualization, Investigation, Conceptualization, Methodology; PC: Review and, Editing, Visualization, Conceptualization, Methodology; AR: Visualization, Resources; EL: Original Draft, Visualization, Resources; ED: Visualization, Resources; WS: Visualization, Resources; BK: Visualization, Resources; RM: Visualization, Resources; AY: Visualization, Resources; MF: Visualization, Resources; DK: Visualization, Resources; DL: Visualization, Resources; NP: Visualization, Conceptualization, Methodology, Resources, Review and, Editing; LR: Visualization, Conceptualization, Methodology, Resources, Review and, Editing. ISG Visualization, Resources. All authors contributed to the article and approved the submitted version.

## Conflict of interest

PC declares research funding from Amgen, AstraZeneca, Merck, Novartis, Roche, Takeda, and advisory board/lecture fees from AstraZeneca, Boehringer Ingelheim, Chugai, Daiichi Sankyo, Gilead, Novartis, Pfizer, Roche, Takeda. NP declares Advisor & Honorarium from & Research with AstraZeneca, Bayer, Boehringer Ingelheim, Bristol-Myers Squibb, Eli Lilly, Foundation Medicine, Gaurdant360, Merk, MSD, Novartis, NovellusDx, Pfizer, Roche, Takeda. WK declares lecture fees from Bristol-Myers Squibb and MSD. ED reported grants from Astra Zeneca, Boehringer Ingelheim, advisory board/lecture fees from Boehringer Ingelheim, Roche, Astra Zeneca, Pfizer, Merck Sharpe & Dohme, Bristol Myers Squibb, Novartis, Takeda, Sanofi, Merck Serono, Medison Pharma, Janssen Israel- all outside of the submitted work. WS declares grants from Astra Zeneca and lecture fees from Merck, Novartis, Roche, Bristol-Myers Squibb, Pfizer and MSD.

The remaining authors declare that the research was conducted in the absence of any commercial or financial relationships that could be construed as a potential conflict of interest.

## Publisher’s note

All claims expressed in this article are solely those of the authors and do not necessarily represent those of their affiliated organizations, or those of the publisher, the editors and the reviewers. Any product that may be evaluated in this article, or claim that may be made by its manufacturer, is not guaranteed or endorsed by the publisher.
